# Countercurrent chromatographic fractionation followed by gas chromatography/mass spectrometry identification of alkylresorcinols in rye

**DOI:** 10.1007/s00216-020-02980-3

**Published:** 2020-10-10

**Authors:** Tim Hammerschick, Tim Wagner, Walter Vetter

**Affiliations:** Department of Food Chemistry (170b), Institute of Food Chemistry, University of Hohenheim, 70599 Stuttgart, Germany

**Keywords:** Alkylresorcinols, Rye, Countercurrent chromatography, Gas chromatography, Mass spectrometry

## Abstract

Alkylresorcinols (5-alkyl-1,3-dihydroxybenzenes, ARs) are bioactive phenolic lipid compounds which are particularly abundant in rye and partly other cereals. In this study on ARs, whole rye grain extracts were gained with cyclohexane/ethyl acetate (46/54, w/w). Silylated extracts were used to develop a gas chromatography with mass spectrometry method in the selected ion monitoring mode (GC/MS-SIM) for the sensitive detection of conventional ARs along with keto-substituted (oxo-AR) and ring-methylated ARs (mAR) with 5-alkyl chain lengths of 14 to 27 carbon atoms and 0 to 4 double bonds in one run. Analysis was performed by countercurrent chromatographic (CCC) fractionation using the solvent system *n-*hexane/ethyl acetate/methanol/water (9/1/9/1, v/v/v/v). Subsequent GC/MS-(SIM) analysis of 80 silylated CCC fractions enabled the detection of 74 ARs in the sample. The CCC elution of the ARs followed the equivalent chain length (ECL) rule in which one double bond compensated the effect of two (additional) carbon atoms. Novel or rarely reported ARs were detected in virtually all classes, i.e. saturated AR (AR14:0), even-numbered monounsaturated AR isomers (AR16:1-AR26:1), triunsaturated ARs (AR25:3), oxo-ARs (AR17:0 oxo, AR19:1 oxo, AR21:2 oxo, AR23:2 oxo) and odd-numbered methyl-ARs (mAR15:0-mAR23:0). Positions of the double bonds of monounsaturated ARs and oxo-ARs were determined with the help of dimethyl disulfide (DMDS) derivatives.

**Electronic supplementary material:**

The online version of this article (10.1007/s00216-020-02980-3) contains supplementary material, which is available to authorized users.

**Figure:**
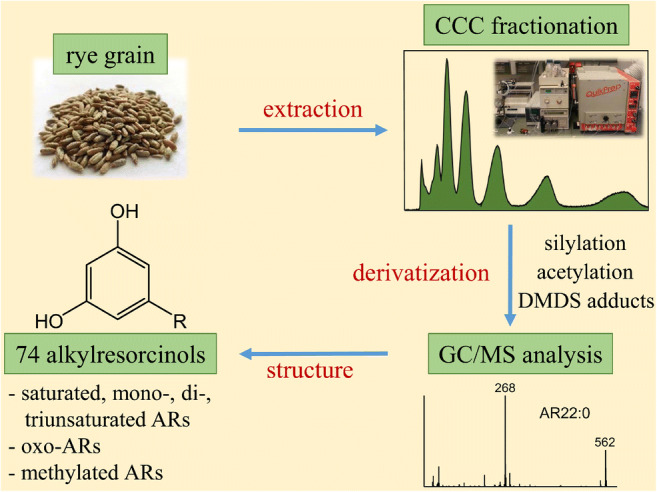
Graphical abstract

## Introduction

**Fig. 1:**
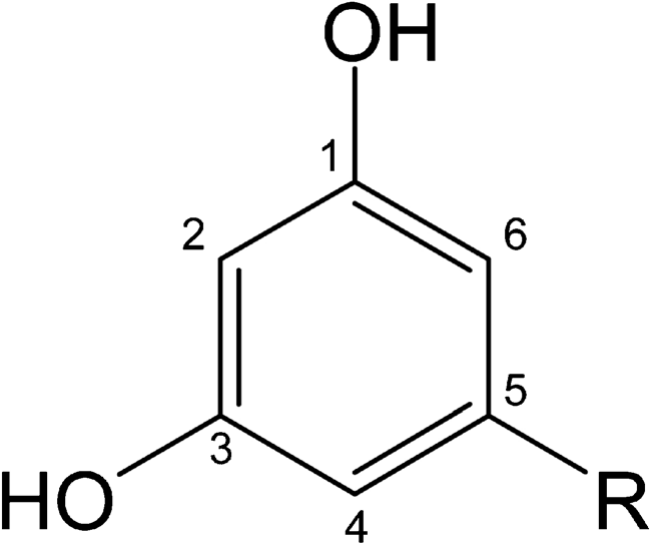
Chemical structure of 5-*n*-alkylresorcinol with *R* = (un)saturated hydrocarbon chain (with keto- or hydroxyl-group)

Alkylresorcinols (ARs) are amphiphilic 1,3-dihydroxybenzene (resorcinol) derivatives characterized by a hydrocarbon chain on C-5 of the resorcinol backbone (Fig. 1) [1]. ARs are bioactive molecules, which may display favourable antioxidative, anticancerogenic, antimicrobial and antiparasitic effects [2, 3]. Also, inhibition of some metabolic enzymes has been observed in in vitro assays [4].

The most relevant dietary sources of ARs are rye and other members of the *Gramineae* family (especially wheat and triticale), in which ARs may contribute with more than 500 μg to 1 g of the fresh weight [1]. Specifically, highest AR amounts are found in the outer cuticula [5]. Rye is not only the richest source of ARs but also known for a high variety of AR structures [1]. In the following, these will be discussed by means of short terms of the “ARn:m” type, in which *n* represents the length of the hydrocarbon chain on C-5 and *m* the number of double bonds.

High-performance liquid chromatography with tandem mass spectrometry (HPLC/MSMS) [6] and gas chromatography with mass spectrometry (GC/MS) analyses [7] showed that *~* 85% of the ARs in rye feature a saturated alkyl chain (ARn:0) [8]. The most relevant representatives are odd-numbered (AR17:0-AR25:0) along with low shares of AR15:0 and AR27:0 and even-numbered homologues (AR16:0-AR24:0) [6, 9]. The remaining share of ~ 15% constitutes mainly of mono- (ARn:1), di- (ARn:2) and tri- (ARn:3) unsaturated hydrocarbon chains (alkenylresorcinols). In addition, hydroxyl (ARn:1-OH) and keto (ARn:0 oxo) groups can be present as substituents on the alkyl chain [6, 7, 10].

The goal of our study was to expand the array of methods for studying AR profiles by means of countercurrent chromatography (CCC). This preparative all-liquid chromatography technique is frequently used in the field of natural and synthetic products [11, 12]. In CCC, the separation is based on the different distribution of the analytes in two immiscible liquid phases, with one being used as stationary and the other one as mobile phase [11]. ARs were not studied by CCC but Marchal et al. developed a strategy for the optimization of the injection step using the related centrifugal partition chromatography (CPC) technique [13]. In that paper, one example included the purification of major ARs with the solvent system *n*-heptane/methanol but without presenting details of the separation [13]. Moreover, a symposium abstract indicated the analysis of an AR extract by CPC [14]. The focus of the present work was to select a suitable solvent system which provides partition coefficients (*K* values) of ARs in the sweet spot range of 0.4 < *K* < 2.5 [15], followed by its use for the analysis of major and minor ARs in rye. For this purpose, small CCC fractions were collected and analysed after silylation by GC/MS measurements. This approach was found to enable the detection of more compounds than without implementing CCC [16]. For example, 430 fatty acid methyl esters were detected in a transesterified butter sample [17] and > 170 tocochromanol artefacts in a vitamin E capsule [18]. In addition, a GC/MS method in the selected ion monitoring (SIM) mode was developed for the detection of minor ARs. The position of the double bond was determined if unknown or confirmed if known by means of dimethyl disulfide (DMDS) derivatives similarly to approaches used for fatty acids and other compounds with double bonds [19, 20].

## Materials and methods

### Rye sample and chemicals

Several 1-kg packages of whole rye grains from German organic cultivation were bought in a retail shop in Stuttgart, Germany. Acetic anhydride (> 99%), anhydrous benzotrifluoride (BTF, > 99%), dimethyl disulfide (> 99%), ethyl acetate (> 99%), pyridine (> 99%, distilled before use) and silica gel 60 were from Sigma-Aldrich (Steinheim, Germany) while methanol, *n*-hexane (both HPLC grade), cyclohexane (> 99%) and toluene (> 99%) were from Th. Geyer (Renningen, Germany). Iodine (> 99.5%) was from Fluka (Taufkirchen, Germany) and sodium thiosulfate (> 99%) was from Carl Roth (Karlsruhe, Germany). The azeotropic mixture cyclohexane/ethyl acetate (CE) (46:54, w/w) was obtained by distillation of mixture (1:1, v/v). N,O-bis(trimethylsilyl)trifluoroacetamide (BSTFA) and trimethylchlorosilane (TMCS), 99:1 (v/v), was from Macherey Nagel (Düren, Germany). The internal standard 5α-cholestane (> 98%) was from Acros Organics (Geel, Belgium) and docosanoic acid methyl ester (22:0-ME) was prepared by the methylation protocol of Wendlinger et al. [21] using 40 mg 22:0 (Fluka, Taufkirchen, Germany) and 4 mL methanol. Helium and hydrogen (5.0 quality) were from Westfalen (Münster, Germany). Demineralized water was produced in-house.

### Extraction of alkylresorcinols from the rye sample

Different solvents including alkanes (*n*-hexane and cyclohexane) and ethyl acetate were suggested for the extraction of different groups of ARs [22]. Own experience with the azeotropic mixture of cyclohexane and ethyl acetate [23] indicated that this solvent mixture could take advantage of the reported positive properties of its individual parts. Accordingly, whole rye grains (60 g for column chromatography, 700 g for CCC) were placed in a 250-mL conical flask or in a 2.5-L brown glass bottle, respectively. The azeotropic CE mixture (100 mL for column chromatography, 1 L for CCC) was added and ARs were cold extracted with occasional shaking for 24 h at room temperature. The resulting raw extracts were passed through a folded filter into a second 250-mL or 1-L flask. The solvent was evaporated by rotary evaporation and the residue was transferred with CE into a pre-weighed 4-mL vial. The weight of the vial was determined after drying using a gentle stream of nitrogen at 40 °C.

### Preparation of trimethylsilyl ether derivatives of alkylresorcinols

Silylation of aliquots of samples and fractions was performed in 1.5-mL vials according to Hammann et al. [24]. The solvent was evaporated under nitrogen at 40 °C. To the dry residue, 50 μL silylating agents (BSTFA/TMCS, 99:1, v/v) and 25 μL pyridine were added and the closed vial was carefully shaken and then heated for 30 min to 60 °C. Afterwards, the excess reagent was evaporated under a gentle stream of nitrogen at 40 °C and the residue was re-dissolved in 1 mL internal standard solution, that is 5 μg/mL 5α-cholestane in *n*-hexane (in the case of the rye extract, all solid phase extraction (SPE) fractions 1–3, shake flask experiments and CCC fractions measured in full scan mode) or 5 μg/mL 22:0-ME in *n*-hexane (in the case of SPE fraction 4 and all CCC fractions and measured in GC/MS-SIM mode). These solutions were used for GC/MS measurements. Noteworthy, silylation takes place in both 1- and 3-positions of ARs (disilylated-ARs). However, for reasons of simplicity, the corresponding products will be listed simply as silylated ARs later on.

### Preparation of acetylated alkylresorcinol derivatives

Aliquots (~ 0.1 mg) of selected CCC fractions (6, 7, 10, 16, 24) were placed in a 1.5-mL vial and supplemented with 50 μL acetic anhydride and 100 μL pyridine [18]. The sealed vial was heated to 60 °C for 2 h and at the end of the reaction, the solvent was removed by a gentle stream of nitrogen at 40 °C. The residue was dissolved in 1 mL internal standard solution (5 μg/mL 5α-cholestane in *n*-hexane or 5 μg/mL 22:0-ME in *n*-hexane).

### Preparation of dimethyl disulfide adducts of alkylresorcinols

Samples were prepared according to the protocol of Buser et al. [20] with slight modifications. An aliquot (*~* 200-μg sample of selected CCC fractions 6, 7, 10, 14, 16, 22, 24, 38 and 59, respectively) was dissolved in 50 μL *n*-hexane in a 2-mL vial. After addition of 100 μL DMDS and 10 μL iodine solution (6% iodine in diethyl ether), the vial was sealed with a cap and heated to 60 °C for 24 h [20]. After cooling, 1 mL aqueous sodium thiosulfate (5%) and 500 μL *n*-hexane were added. The vial was shaken and after phase separation, 300 μL of the upper organic phase was carefully transferred into a new vial. The solvent was evaporated and the residue was silylated and analysed by GC/MS.

### Solid phase extraction in a glass column

Lipid classes were separated by SPE in duplicate according to Hammann et al. [25]. Five grams of deactivated silica gel (20% water, w/w) was placed in a glass column (1-cm inner diameter). After conditioning of the column with 30 mL *n*-hexane, the AR extract (~ 100 mg dissolved in 2 mL *n*-hexane) was placed onto the column. SPE fraction 1 containing hydrocarbons was eluted with 30 mL *n*-hexane. Steryl esters (SPE fraction 2) were eluted with 40 mL *n*-hexane/ethyl acetate (99:1, v/v). SPE fraction 3 (triacylglycerols) was obtained with 50 mL *n*-hexane/ethyl acetate (95:5, v/v). Finally, ARs, free fatty acids, sterols and other alcohols were gained by flushing the column with 40 mL ethyl acetate (SPE fraction 4). Each SPE fraction was collected in a separate 100-mL pear-shaped flask and the solvent was evaporated to dryness by rotary evaporation. The residues were transferred with *n*-hexane into pre-weighed vials, the solvent was evaporated under a gentle stream of nitrogen at 40 °C and the vials were weighed again. An aliquot of 100 μg of each SPE fraction was silylated and analysed by GC/MS.

### Determination of *K*_U/L_ values for alkylresorcinols

To find a suitable solvent system for the CCC separation, shake flask tests according to Ito [11] were performed with the setup of Schröder et al. [26] except the use of slightly larger sample vials. About 200 μg of the rye extract eluting into SPE fraction 4 was placed into an 8-mL vial and 1 mL of the lower and upper phase of the pre-equilibrated solvent systems was added. After vigorous shaking and phase separation, aliquots of 500 μL were transferred from both phases into distinct 1.5-mL vials. After removing the solvents from both vials by a gentle stream of nitrogen at 40 °C, ARs were silylated as described above. Then, the solutions were evaporated again to dryness and made up with exactly 1 mL *n*-hexane supplemented with 5 μg of the internal standard 5α-cholestane. GC/MS peak areas of individual ARs were normalized by the peak area of the internal standard 5α-cholestane in the respective solution. GC/MS measurements were carried out in triplicate and mean *K*_U/L_ values were calculated from the corrected peak areas by dividing the peak areas of the substances in the upper phase by the complementary peak areas in the lower phase.

### Countercurrent chromatography

CCC separations were performed with a Quickprep MK8 instrument (AECS London, UK) using the periphery recently described by Hammann et al. [24]. Two of the instrument’s four coils, namely coil 2 in bobbin 1 and coil 3 in bobbin 2 (total volume 236 mL), were selected for the separations. The rotor speed was set to the maximum value of 870 rpm. The temperature was maintained at 22 °C by external cooling. The effluent of the CCC centrifuge was continuously monitored at a wavelength of 210 nm with a flash 10 diode array detector (DAD; Ecom, Praha, Czech Republic) and fractions were collected using a Gilson 203 B fraction collector (Middleton, WI, USA).

The solvent system *n*-hexane/ethyl acetate/methanol/water (9:1:9:1, v/v/v/v) was prepared in a 2.5-L separation funnel. After vigorous shaking and an equilibration time of 1 h, both phases were separated and degassed by ultrasonication. The selected coils were filled with upper (stationary) phase at 10 mL/min. Thereafter, the flow rate was reduced to 2 mL/min and rotation was started. In head-to-tail mode, the lower (mobile) phase was pumped into the two coils. After determination of the retention of the stationary phase (*S*_f_ = 86%, 33-mL extruded stationary phase) by collecting the effluent in a graduated cylinder, 900 mg of the rye extract, dissolved in 4.5 mL lower and 4.5 mL upper phase, was injected into the CCC system. After a delay of 30 mL, 80 fractions of 7 mL each were collected from 30 to 590 mL. CCC fractions were liberated from solvent by means of a rotational vacuum concentrator (10 mbar, 80 °C, 1500 rpm) (RVC 2-33 IR, Martin Christ, Osterode am Harz, Germany). After determination of the sample weight, fractions were re-dissolved in 1 mL CE. Aliquots of each vial were silylated and analysed by GC/MS-SIM.

### Gas chromatography with mass spectrometry

Silylated total lipid extracts, solid phase extraction fractions 1 to 3 as well as solutions with DMDS adducts and acetylated sample solutions were analysed on GC/MS system 1 (6890/5973 GC/MS system equipped with a cool-on-column inlet and a 7683 autosampler, Hewlett-Packard/Agilent, Waldbronn, Germany). Injections (1 μL) were made onto a Zebron guard column with deactivated tubing (2 m, 0.53-mm i.d., Phenomenex, Aschaffenburg, Germany) connected to a ZB-1HT capillary column (15 m, 0.25-mm i.d., 0.1-μm film thickness, Phenomenex, Aschaffenburg, Germany). Helium (5.0) was used as the carrier gas at a flow rate of 1.2 mL/min. The column oven was programmed according to Hammann et al. [18] but with a higher initial temperature: After 1 min at 100 °C, the oven was heated at 10 °C/min to 250 °C (hold time 5 min), then at 5 °C/min to 300 °C and finally the temperature was raised at 30 °C/min to 350 °C (hold time 10 min). Transfer line (320 °C), ion source (230 °C) and quadrupole (150 °C) were heated to the values shown in parentheses. After a solvent delay of 6 min, data was recorded from *m*/*z* 50 to 800 in GC/MS full scan mode.

Silylated CCC fractions and SPE fraction 4 (containing ARs) were analysed with GC/MS system 2 (a second 6890/5973 GC/MS system equipped with split/splitless injector (Hewlett-Packard/Agilent, Waldbronn, Germany). An MPS 2 autosampler (Gerstel, Mülheim, Germany) was used for splitless injections (1 μL) into the injector port maintained at 250 °C. The carrier gas helium 5.0 with a constant flow rate of 1.0 mL/min was transported through a Zebron guard column with deactivated tubing (2 m, 0.25-mm i.d.) (Phenomenex, Aschaffenburg, Germany) followed by an Optima 5HT column (30 m, 0.25-mm i.d., 0.25-μm film thickness, Macherey Nagel, Düren, Germany). After 1 min at 55 °C, the GC oven was heated to 200 °C with a ramp of 10 °C/min (no hold time), and finally the oven temperature was raised at 5 °C/min to 320 °C (15-min hold time). Temperatures of the transfer line, ion source and quadrupole were set at 280 °C, 230 °C and 150 °C, respectively. In the full scan mode, *m*/*z* 50–650 were measured over the total run time of 54.5 min after a solvent delay of 7 min.

GC/MS-SIM measurements were applied to possible ARs with chain lengths of 14 to 27 carbon atoms and 0 to 4 double bonds (see Electronic Supplementary Material (ESM), Table S1). GC/MS analysis of silylated extracts allowed a secure identification of prominent ARs based on the distinct molecular ions [27] along with three additional diagnostic fragment ions. These are (i) the base peak at *m*/*z* 268 (formed by McLafferty rearrangement of the resorcinol ring) along with (ii) the ditrimethylsilyloxy substituted tropylium ion at *m*/*z* 267 (formed by benzyl cleavage) and (iii) *m*/*z* 281 (formed by cleavage between C2 and C3 in the alkyl chain) (ESM Fig. S1) [28, 29].

## Results and discussion

### GC/MS analysis of rye grain extract

**Fig. 2:**
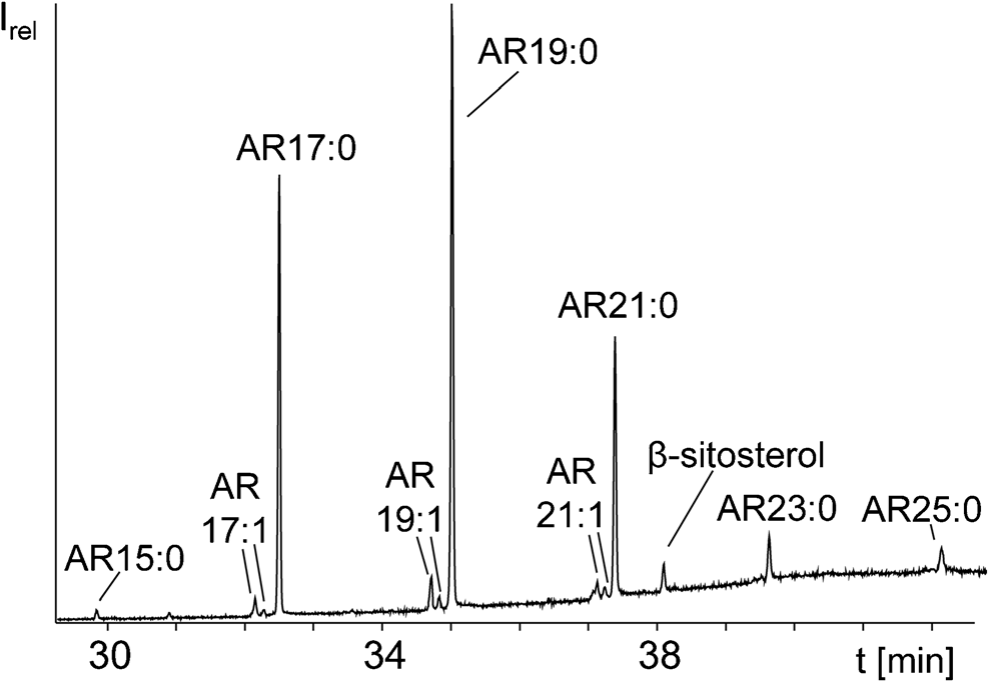
GC/MS chromatogram (full scan) of the silylated content of fraction 4 after column chromatography of rye grain extract (system 2, Optima 5HT, 55 °C (1 min) – 10 °C/min – 200 °C – 5 °C/min – 320 °C (15 min))

Evaporation of the solvent after cold extraction of 700-g whole rye grain for CCC separation yielded 2.54-g sample (0.36%). GC/MS analysis of a silylated aliquot verified the predominance of odd-numbered ARs AR15:0-AR25:0 along with odd-numbered alkenylresorcinols AR17:1-AR21:1 (Fig. 2 and ESM Fig. S2). In addition, the silylated extract featured triacylglycerols as well as silylated β-sitosterol and campesterol (ESM Fig. S2) [8]. SPE fractionation of two 100-mg aliquots of the 0.21-g extract from 60-g whole rye grain according to Hammann et al. [25] showed that all ARs eluted into SPE fraction 4 (49.5% of sample mass), together with low amounts of β-sitosterol (Fig. 2), campesterol (which co-eluted with AR21:1) and traces of free fatty acids. The remaining mass originated from triacylglycerols (SPE fraction 3, 45.9% of sample mass), hydrocarbons (SPE fraction 1, 0.8% of sample mass) and steryl esters (SPE fraction 2, 3.8% of sample mass), so that their separation from ARs could be achieved. A closer inspection of the AR-containing SPE fraction 4 (Fig. 2) indicated the presence of ~ 90% saturated ARs and ~ 10% alkenylresorcinols (based on peak areas). The pattern of saturated ARs, namely AR15:0 (1%), AR17:0 (26%), AR19:0 (37%), AR21:0 (22%), AR23:0 (8%) and AR25:0 (6%), agreed well with the reported ranges for some rye species (AR17:0, 21–26%; AR19:0, 30–35%; AR21:0, 23–28%; AR23:0, 8–12% and AR25:0, 6–9%) [8]. Further minor ARs were detected in form of monounsaturated ARs which eluted slightly earlier than the corresponding saturated ARs from the GC column (Fig. 2). Their partly low abundance and low S/N ratio prompted us to implement a GC/MS-SIM method which should cover ARs with chain lengths of 14 to 27 carbon atoms and 0 to 4 double bonds. The required 70 molecular ions (M^+^) were screened in 16 time windows implemented based on the retention times of major ARs (ESM Table S1). Likewise, the three characteristic fragment ions *m*/*z* 267.1, 268.1 and 281.1 of silylated ARs [28, 29] and the diagnostic base peak of silylated ring-methylated ARs (mARs) at *m*/*z* 282.1 (+ 14 u compared *m*/*z* 268.1 of silylated ARs) [30] were measured throughout the run (ESM Table S1). ARs with one keto group (oxo-ARs) were also covered by the GC/MS-SIM method because they are isobaric with conventional ARs having one carbon atom more, irrespective of the degree of saturation (ESM Table S1). For instance, AR21:0 oxo and AR22:0 as well as AR23:1 oxo and AR24:1 share the same M^+^, respectively. To establish the individual time windows, it was considered that isobaric ARs eluted in the order mAR < AR < oxo-AR [7, 30] and unsaturated ARs eluted between mAR and saturated ARs (mAR < unsaturated ARs < saturated AR < oxo-AR; AR groups with the same molecular ion range). The resulting GC/MS-SIM method enabled the additional detection of low amounts of AR27:0, AR 27:1, AR17:2, AR19:2 and even-numbered ARs (AR16:0-AR 22:0) along with traces of AR17:0 oxo, AR19:0 oxo, AR21:0 oxo and AR23:0 oxo in the silylated SPE fraction 4 of the lipid class separation. By using this sensitive GC/MS-SIM method, the number of detected ARs could be more than doubled (namely 29 ARs by GC/MS-SIM versus 11 ARs by GC/MS in full scan mode). Moreover, SPE fraction 4 was dominated of ARs, so that it could directly be used for the determination of *K* values in shake flask experiments in order to prepare a successful CCC separation.

### Selection of a suitable solvent system for the CCC fractionation of alkylresorcinols by means of the shake flask method

The high logarithmic octanol/water partition coefficients (log *P* values) of saturated ARs (log *P*, AR15:0 = 8.5, AR25:0 = 13.4 [31]) are typical of nonpolar compounds and spread over five orders of magnitude. Hence, saturated ARs in the sample obtained after column chromatography were used to determine the *K* values in five solvent systems. Similar to observations with heptane/methanol [13], *n*-hexane/methanol/water (10:91:9, v/v/v) partitioned ARs mainly into the lower phase (Table 1). Switching to *n*-hexane/acetonitrile (1:1, v/v) even enhanced this effect because *K*_U/L_ values (0.06–0.55) were roughly halved (Table 1). Addition of the modifier benzotrifluoride in form of the BTF system (*n*-hexane/benzotrifluoride/acetonitrile 20/7/13, v/v/v, [32]) increased the *K*_U/L_ values (Table 1). However, all of them were in the low range (*K*_U/L_ < 0.75) and *α* values indicated an insufficient resolution. Interestingly, substitution of BTF with toluene as a modifier (*n*-hexane/toluene/acetonitrile, 45:10:45, v/v/v [33]) provided smaller *K*_U/L_ values for shorter ARs and larger *K*_U/L_ values for longer ARs (Table 1). Still this solvent system was unsuited for a good AR separation, especially in the range of the shorter alkyl lengths (AR15:0-AR19:0) (Table 1). By contrast, application of the moderately nonpolar member of the HEMWat family, *n*-hexane/ethyl acetate/methanol/water (7:3:6:4, v/v/v/v) (HEMWat-4 [34]) distributed the ARs almost exclusively into the upper phase (*K*_U/L_ ~ 100) (Table 1). However, the least polar and stable solvent system of the HEMWat family, *n*-hexane/ethyl acetate/methanol/water (9:1:9:1, v/v/v/v, HEMWat -7), generated favourable and unique *K*_U/L_ values ranging from 0.26 to 2.35 for major ARs in rye.

**Table 1:** Partitioning coefficients between the upper and lower phase (*K*_U/L_ values) of odd-numbered ARs (AR15:0 to AR25:0) determined in shake flask experiments and GC/MS for six solvent systems (mean values after triplicate)

	Hex/EtOAc/MeOH/H_2_O (7:3:6:4)	Hex/ACN (1:1)	Hex/BTF/ACN (100:35:65)	Hex/Tol/ACN (45:10:45)	Hex/MeOH/H_2_O (100:91:9)	Hex/EtOAc/MeOH/H_2_O (9:1:9:1)
AR15:0	~ 100	0.06	0.34	0.16	0.09	0.26
AR17:0	~ 100	0.08	0.37	0.18	0.16	0.35
AR19:0	~ 100	0.10	0.42	0.25	0.20	0.52
AR21:0	~ 100	0.17	0.51	0.39	0.37	0.90
AR23:0	~ 100	0.33	0.58	0.69	0.79	1.71
AR25:0	~ 100	0.55	0.71	1.18	1.46	2.35

According to Berthod et al., the partition coefficients of a homologous family (like ARs) are linked to the alkyl chain carbon number *nC* by Eq. 1 [35]:

1 $$ \log\ K=A\times nC+B $$

with *K*, the *K* value of the AR with alkyl chain carbon number *nC*; *A*, the coefficient related to the ∆*G* of methylene transfer between the two liquid phases (∆*G = −RT* log *K*); and *B*, the resorcinol *K* value in the considered system.

The *K* values determined by experiments showed acceptably well-aligned log *K* values with *nC*, which were in agreement with theoretical considerations according to Eq. 1.

**Fig. 3:**
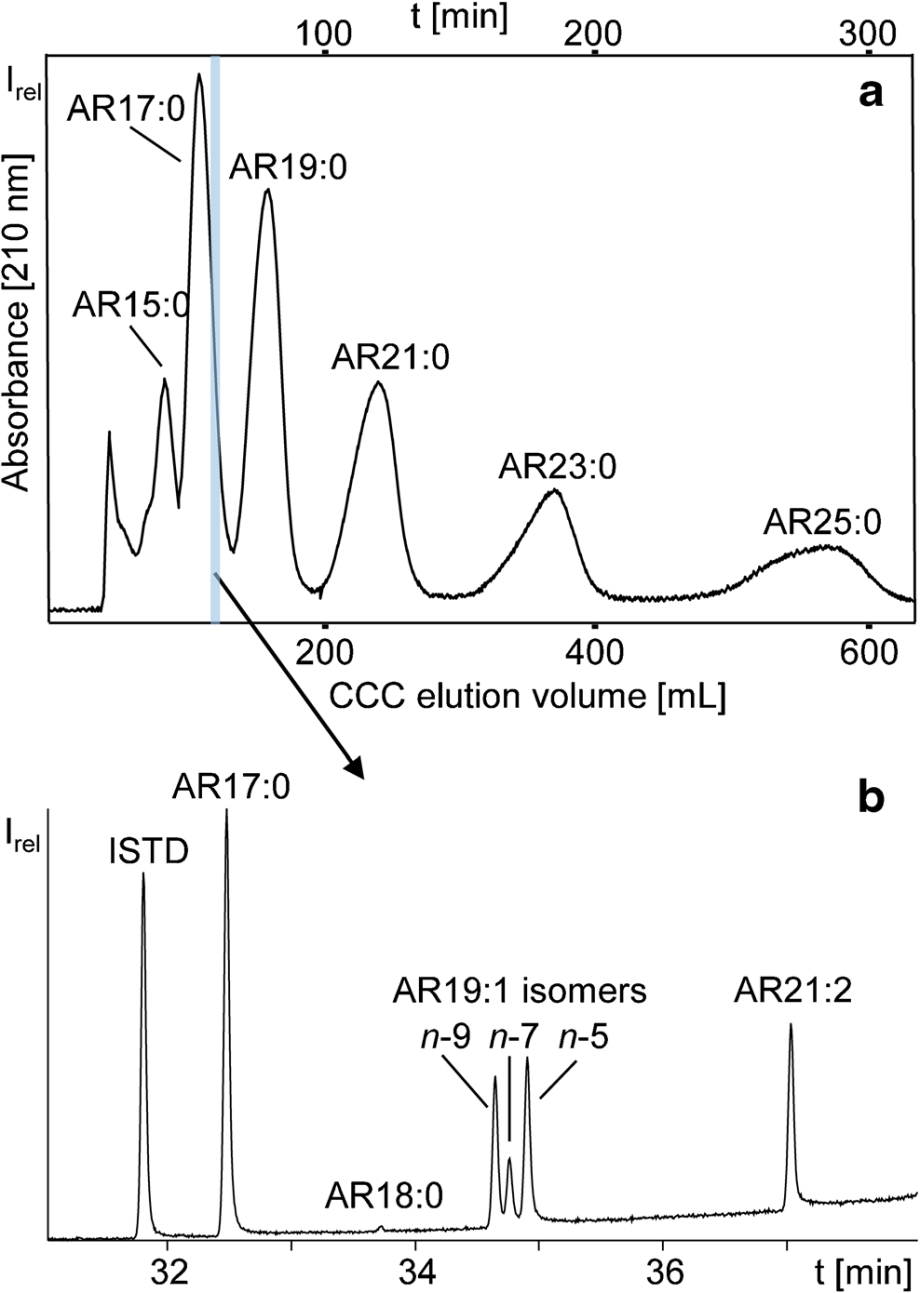
**a** CCC/UV chromatogram (210 nm) of the separation of ARs from rye grain extract with the solvent system *n*-hexane/ethyl acetate/methanol/water (9:1:9:1, v/v/v/v). Separation was performed in head-to-tail mode at a flow rate of 2 mL/min, with *S*_f_ = 86%, coil volume of 236 mL and 22 °C. **b** GC/MS chromatogram (full scan) of fraction 14 collected from 121 to 128 mL (EV 37.3–40.3%) after silylation (system 2, Optima 5HT, 55 °C (1 min) – 10 °C/min – 200 °C – 5 °C/min – 320 °C (15 min))

The software “ProMISE 2” [36] was used to predict the most appropriate number of coils to be used in the separation (1–4 coils of ~ 120 mL can be freely selected with our CCC [37]). Accordingly, the use of two coils (236 mL) was found to be appropriate (ESM Fig. S3b), because this setup indicated a good separation of all major ARs (contrary to one 120-mL coil) (ESM Fig. S3a) within reasonable time (contrary to three (352 mL) (ESM Fig. S3c) or four coils). Also less and smaller CCC fractions could be collected with two coils being used. Using this setup, the *K*_U/L_ values corresponded with calculated elution volumes of 60–560 mL (Fig. 3). Last but not least, this range was fully compatible with our instrument’s fraction collector which allows collecting up to 80 fractions with up to 7 mL each. Hence, 80 7-mL fractions were collected from 30 to 590 mL.

### CCC separation and GC/MS-SIM analysis of the silylated fractions

**Fig. 4:**
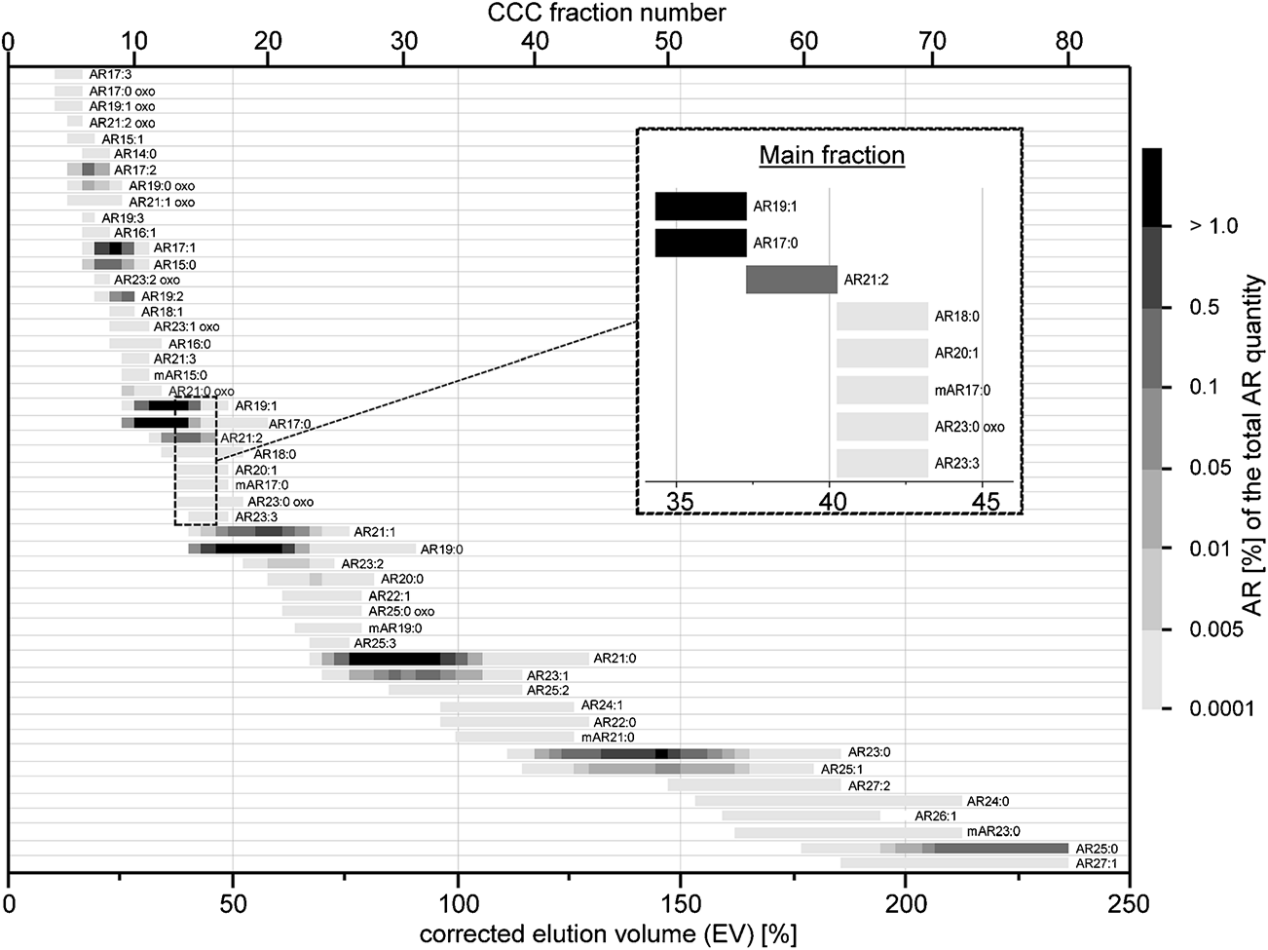
Corrected CCC elution volume of the ARs from rye grain after separation with the solvent system *n*-hexane/ethyl acetate/methanol/water (9:1:9:1, v/v/v/v) in head-to-tail mode, 2 mL/min flow rate and *S*_f_ = 86%

The CCC-UV chromatogram (210 nm) obtained from the injection of a 900-mg aliquot of the whole rye extract showed six distinct peaks whose elution volumes were in agreement with those simulated for the odd-numbered AR15:0 to AR25:0 (Fig. 3a vs. ESM Fig. S3b). However, GC/MS-SIM inspection of individual fractions enabled the detection of more than one AR per fraction. For instance, CCC fraction 14 (121–128 mL) not only featured AR17:0 but also AR19:1 isomers, AR21:2 and traces of AR18:0 (Fig. 3a, b). Elution of AR17:0, AR19:1 isomers and AR21:2 into one CCC fraction indicated that presence of a double bond in the alkyl chain of ARs had a similar effect on the CCC elution as two additional carbon atoms (Fig. 4). This relation resembled the effect of double bonds on the elution of fatty acids and their methyl esters according to the so-called equivalent chain length (ECL) rule [38, 39]. Similar rules were also developed for other lipid classes and proved to be helpful for the prediction of co-elutions [40]. Moreover, several AR19:1 isomers were detected by GC/MS which could not be resolved by CCC (Fig. 3).

The full evaluation of the sample was based on the normalization mode according to Hammann et al. which reports results independent of fraction or coil volumes [16] (Eq. 2):

2 $$ \mathrm{EV}=\frac{V_m-{V}_e}{V_c}\times 100\%=\frac{V_m-33\ \mathrm{mL}}{236\ \mathrm{mL}}\times 100\% $$

with EV, the corrected elution volume in percent; *V*_*m*_, the volume of mobile phase; *V*_*e*_, the volume of extruded stationary phase (here 33 mL); and *V*_*c*_, the total coil volume (here 236 mL).

EV values of ARs calculated according to Eq. 2 were in good agreement with *K*_U/L_ values determined by shake flask experiments (when transferred on % scale). To convert the *K*_U/L_ values into their corresponding EV values, the *K*_U/L_ values are simply multiplied by the *S*_f_ value [%] (EV = *K* • *S*_f_). For instance, the (shake flask-derived) *K*_U/L_ value 0.26 of AR15:0 resulted in an EV value of (0.26 • 86% =) 22.4% while its EV value after CCC separation ranged from 16.5 to 31.4% (mean EV 23.9%). In addition to ARs, low amounts of the free fatty acids linoleic acid (18:2*n-*6, EV 132.2–153%), oleic acid (18:1*n*-9, EV 167.8–212.3%) and palmitic acid (16:0, EV 158.9–212.3%) were also detectable in several fractions, but will not be considered further below. ARs were detected from CCC fraction 5 to CCC fraction 80 (end of fractionation) (Fig. 4). As anticipated, CCC fractionation enabled the detection of further, partly uncommon/unknown ARs than without CCC. For a better description, ARs will be discussed in groups.

### Alkylresorcinols with saturated alkyl chains

In head-to-tail mode (lower phase mobile), saturated ARs eluted with increasing chain length (Fig. 4). The elution volumes of the individual saturated compounds increased exponentially according to Eq. 1 for the relationship of the homologous compounds (Fig. 4). Odd-numbered (classic) saturated ARs (AR15:0-AR25:0) were detected in almost all CCC fractions (7–80, EV 16.5–236%) and were the dominant ARs. For instance, AR17:0, AR19:0 and AR23:0 together accounted for ~ 75% of the total AR content (Table 2). Shorter or longer odd-numbered ARs were not detected. However, the sensitive GC/MS-SIM method enabled the detection of even-numbered ARs even in the ppm range (0.0001–0.0232% share of the alkylresorcinols). Even-numbered ARs eluted between the respective odd-numbered representatives. Besides the known even-numbered AR16:0-AR24:0 in rye, AR14:0 (CCC fractions 6–8) was detected in rye for the first time. Accordingly, AR14:0 represented the AR with the shortest alkyl chain in the rye sample.

**Table 2:** Alkylresorcinols in rye grains identified by GC/MS-SIM analysis after CCC fractionation

Alkylresorcinol	GC retention time (min)	Contribution to total alkylresorcinols	CCC fractions	Main fraction	CCC elution order^a^	Reference
Saturated		85.3%				
AR14:0	28.48	tr^b^	6–8	7	6	
AR15:0	29.86	0.8%	7–11	8	13	[6, 8, 9]
AR16:0	31.19	tr^b^	9–12	10	18	[6]
AR17:0	32.50	23.0%	10–20	13	23	[6, 8, 9]
AR18:0	33.77	tr^b^	13–18	15	25	[6]
AR19:0	35.00	27.7%	15–31	20	31	[6, 8, 9]
AR20:0	36.21	tr^b^	21–28	24	33	[6]
AR21:0	37.38	24.4%	24–44	31	38	[6, 8, 9]
AR22:0	38.51	tr^b^	34–44	37	42	[6]
AR23:0	39.62	6.5%	39–72	50	44	[6, 8, 9]
AR24:0	40.80	tr^b^	54–72	60	47	[6]
AR25:0	42.18	2.9%	61–80	78	50	[6, 8, 9]
Monounsaturated^c^		13.6%				
AR15:1	29.60 (*n-7*), 29.76 (*n-5*)	tr^b^	6–7	7	5	
AR16:1	30.76 (*n-9*)	tr^b^	7–8	8	11	
AR17:1	32.17 (*n-9*), 32.29 (*n-7*), 32.45 (*n-5*)	3.0%	7–11	10	12	[6, 41]
AR18:1	33.45 (*n-9*), 33.55 (*n-7*)	tr^b^	9–10	10	16	
AR19:1	34.73 (*n-9*), 34.84 (*n-7*), 34.98 (*n-5*)	6.3%	10–17	13	22	[6, 41]
AR20:1	35.93 (*n-9*), 36.14 (*n-7*)	tr^b^	14–17	15	26	
AR21:1	37.13 (*n-9*), 37.25 (*n-7*), 37.38 (*n-5*)	3.0%	15–26	20	30	[6, 41]
AR22:1	38.29 (*n-9*), 38.39 (*n-7*)	tr^b^	22–27	23	34	
AR23:1	39.42 (*n-9*), 39.52 (*n-7*), 39.63 (*n-5*)	0.9%	25–39	33	39	[6, 41]
AR24:1	40.57 (*n-9*), 40.68 (*n-7*)	tr^b^	34–43	39	41	
AR25:1	41.90 (*n-9*), 42.02 (*n-7*), 42.16 (*n-5*)	0.4%	40–61	51	45	[6]
AR26:1	43.40 (*n-9*)	tr^b^	55–68	59	48	
AR27:1	45.16 (*n-9*), 45.30 (*n-7*), 45.50 (*n-5*)	tr^b^	64–80	79	51	[6]
Diunsaturated		1.0%				
AR17:2	32.13	0.2%	6–8	7	7	[6]
AR19:2	34.70	0.3%	8–10	10	15	[6, 41]
AR21:2	37.14	0.5%	12–16	14	24	[6, 41]
AR23:2	39.43	tr^b^	19–25	22	32	[6, 41]
AR25:2	41.91	tr^b^	30–39	34	40	[6, 41]
AR27:2	45.17	tr^b^	51–63	56	46	[6]
Triunsaturated						
AR17:3	32.24	tr^b^	5–6	5	1	[6]
AR19:3	34.82	tr^b^	7	7	10	[6]
AR21:3	37.26	tr^b^	10–11	11	19	[6]
AR23:3	39.55	tr^b^	15–17	16	29	[6]
AR25:3	42.10	tr^b^	24–26	25	37	
mAR						
mAR15:0	30.56	tr^b^	10–11	10	20	
mAR17:0	33.20	tr^b^	14–17	15	27	
mAR19:0	35.69	tr^b^	23–27	24	36	
mAR21:0	38.05	tr^b^	35–43	39	43	
mAR23:0	40.31	tr^b^	56–72	62	49	
AR oxo^c^						
AR17:0 oxo	33.98	tr^b^	5–6	5	2	
AR19:0 oxo	36.45	tr^b^	6–9	7	8	[7]
AR21:0 oxo	38.78	tr^b^	10–12	10	21	[7]
AR23:0 oxo	41.15	tr^b^	14–18	14	28	[7]
AR25:0 oxo	44.16	tr^b^	22–27	25	35	[7]
AR19:1 oxo	36.16 (*n-9*), 36.27 (*n-7*), 36.42 (*n-5*)	tr^b^	5–6	7	3	[10]
AR21:1 oxo	38.53 (*n-9*), 38.64 (*n-7*), 38.76 (*n-5*)	tr^b^	6–9	8	9	[7, 10]
AR23:1 oxo	40.89 (*n-9*), 41.00 (*n-7*), 41.14 (*n-5*)	tr^b^	9–11	11	17	[7, 10]
AR21:2 oxo	38.51	tr^b^	6	6	4	[10]
AR23:2 oxo	40.87	tr^b^	8	8	14	[10]

^a^Numbering based on CCC elution order (Fig. 4)

^b^Trace amounts, contribution < 0.1% to the total alkylresorcinols content

^c^Several isomers; GC retention time indicates the amount of isomers with the position of double bond

### Alkylresorcinols with unsaturated alkyl chains (alkenylresorcinols)

Alkenylresorcinols with one (*n* = 29), two (*n* = 6) and three (*n* = 5) double bonds were detected as well (Table 2). Monounsaturated alkenylresorcinols covered thirteen subsequent chain lengths, including traces of even-numbered ones (AR16:1-AR26:1) and noticeably higher proportions of odd-numbered (AR15:1-AR27:1) members. For instance, members with the predominant chain lengths (AR17:1 (3%), AR19:1 (6.3%) and AR21:1 (3%)) contributed with > 12% to the AR content of the rye sample (Table 2). Three AR27:1 isomers were detected but not the corresponding AR27:0 due to its *K* value outside the fractionation range (*K* > 2.7). However, AR27:0 was detected together with triacylglycerols, β-sitosterol and campesterol in the fraction obtained by elution-extrusion of the complete coil volume (data not shown).

**Fig. 5:**
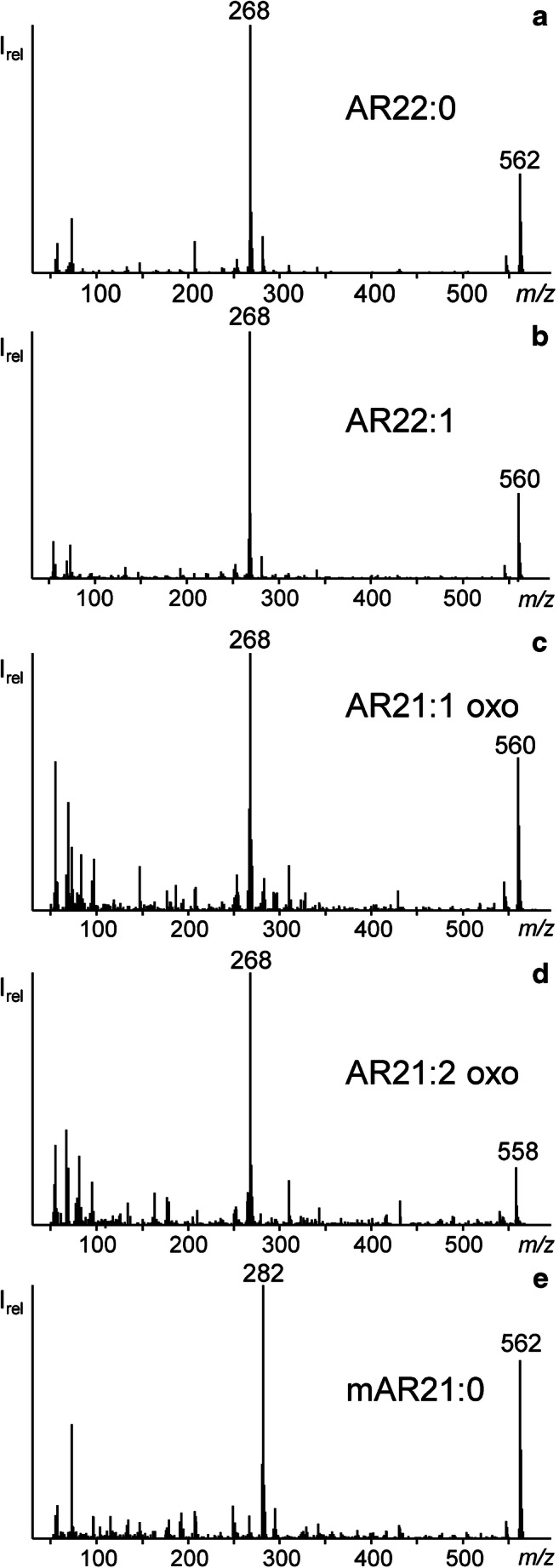
GC/MS spectra of the TMS derivatives of (**a**) AR22:0 (38.52-min retention time), (**b**) AR22:1 (38.39-min retention time), (**c**) AR21:1 oxo (38.76-min retention time), (**d**) AR21:2 oxo (38.51-min retention time) and (**e**) mAR21:0 (38.05-min retention time) with their molecular ion and the typical base ion of *m*/*z* 268 for AR and *m*/*z* 282 for mAR

**Fig. 6:**
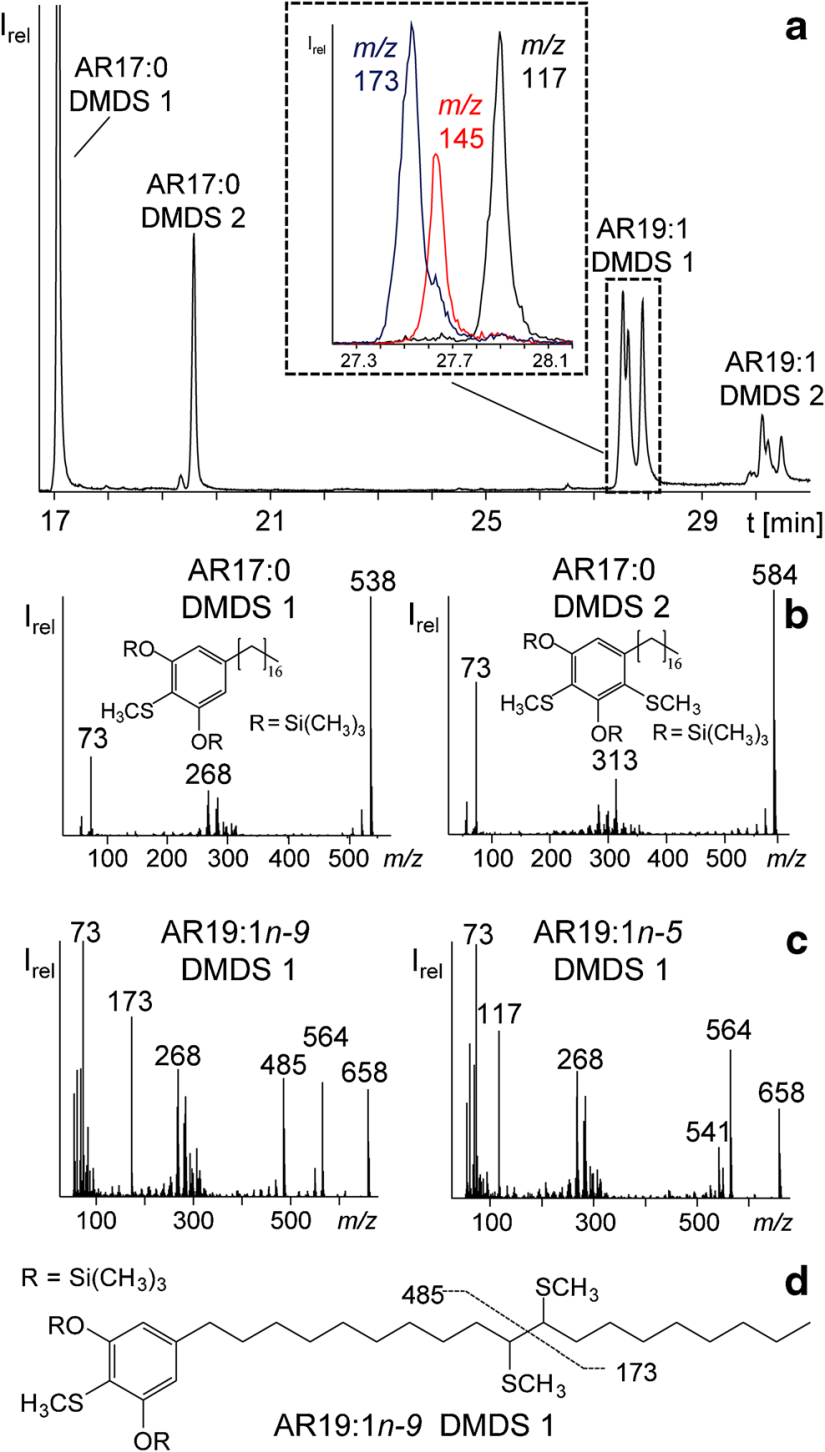
**a** GC/MS chromatogram (full scan) of the DMDS adducts of fraction 14 after CCC separation with a small section of the fragment ions *m*/*z* 173, 145, 117 (system 1, ZB-1HT, 100 °C (1 min) – 10 °C/min – 250 °C (5 min) – 5 °C/min – 300 °C – 30 °C/min – 350 °C (10 min)). **b** Mass spectra of the DMDS adducts (1+2) of AR17:0. **c** Mass spectra of the DMDS adduct 1 of AR19:1*n-9* and AR19:1*n-5*. **d** Structure of AR19:1*n-9* with shown fragmentation

For most monounsaturated ARs, three isomers were found (e.g. AR19:1 (Fig. 3b, Table 2). The *t*_R_ distance and range of monounsaturated ARs in GC was almost the same for all isomer groups (overall Δ*t*_R_ ~ 0.12 min, ESM Fig. S4) which indicated double bonds in characteristic positions. However, the abundance ratio of three peaks was not uniform; i.e., the first, second or third isomer could be the most abundant one, respectively (ESM Fig. S4). In literature, double bonds of odd-numbered monounsaturated ARs (AR17:1-AR23:1) were assigned to *n-5*-, *n-7*- and *n-9*-positions by collision-activated dissociation (CAD) tandem mass spectrometry analysis [41]. However, double bond positions in even-numbered monounsaturated ARs like AR22:1 (Fig. 5) and other minor ARs had not been determined, yet. For this purpose, we prepared DMDS adducts from several CCC fractions in order to confirm known structures and to clarify those of unknown ARs (Table 2). GC/MS chromatograms of derivatized CCC fractions showed two peaks for each AR which differed by ~ 2.6 min in *t*_R_. Noteworthy, two adducts were also observed for saturated silylated ARs (Fig. 6a). As shown by Knödler et al., one or two methyl sulfide groups can also be added to resorcinol moiety [19]. Hence, saturated ARs formed ions representing the substitutions of H by SCH_3_ (added mass 46 u) or 2x H by 2x SCH_3_ (92 u)). Accordingly, the molecular ion of peak 1 was 46 u higher than the corresponding AR (DMDS-mono adduct 1, e.g. M^+^, [TMS-*O*-AR17:0+SCH_3_-H]^+^ = *m*/*z* 538) while the second peak was 92 u higher in mass (DMDS adduct 2, e.g. M^+^, [TMS-*O*-AR17:0+2SCH_3_-2H]^+^ = *m*/*z* 584) (Fig. 6b).

This reaction in the aromatic part was also observed in the case of monounsaturated ARs, along with the addition of two SCH_3_ units in the alkyl chain. Hence, in the case of monounsaturated ARs, three or four SCH_3_ units were added. Therefore, the corresponding peaks were shifted by (46+94) u (adduct 1) and (94+94) u (adduct 2) to higher mass. For instance, after reaction with DMDS and subsequent silylation of AR19:1, the resulting TMS-*O*-AR19:1 (*m*/*z* 518) generated peaks, namely one peak with M^+^ at *m*/*z* 658 ([TMS-*O*-AR19:1+3SCH_3_-H]^+^ or [TMS-*O*-AR19:1+140 u]^+^ (Fig. 6c) and one peak with M^+^ at *m*/*z* 704 ([TMS-*O*-AR19:1+4SCH_3_-2H]^+^ or [TMS-*O*-AR19:1+186 u]^+^). Positions of double bonds can be determined by the fragment ions that were formed during the cleavage of the C–C bond between the CH_3_S substituents in the alkyl chain, with charge on the left hand and the right hand sides, respectively (Fig. 6d) [20]. The nominal *m*/*z* values for these key fragments belong to the series *m*/*z* (61 + *m* • 14) for fragment ions including the terminal part of the alkyl chain ([H(CH_2_)_m_CH=SCH_3_]^+^) [20] and *m*/*z* (359 + *n* • 14) for fragment ions including the head group (i.e. the silylated resorcinol moiety ([CH_3_S=CH(CH_2_)_*n*_C_6_H_2_SCH_3_(OSi(CH_3_)_3_)_2_]^+^) (DMDS adduct 1) and corresponding fragment ions added together result in the molecular ion (e.g. AR19:1*n-9* DMDS adduct 1, *m*/*z* (173 + 485=) 658 (Fig. 6c, d, ESM Table S2).

Accordingly, the most important key fragment ions of monounsaturated ARs with a double bond in *n-9*-, *n-7*- and *n-5*-positions are *m*/*z* 173 (*m*/*z* 61 + 8 • 14), *m*/*z* 145 (*m*/*z* 61 + 6 • 14) and *m*/*z* 117 (*m*/*z* 61 + 4 • 14), respectively (ESM Table S2). These measurements verified the presence of three AR19:1 isomers which eluted in the order (silylated) AR19:1*n-9* < AR19:1*n-7* < AR19:1*n-5* (Fig. 6a). The corresponding three isomers were detected for all odd-numbered monounsaturated AR17:1-AR25:1 except for AR15:1 which lacked the *n-9*-isomer (Table 2). Moreover, the even-numbered monounsaturated ARs featured two isomers with double bonds in *n-7* and *n-9* positions for AR18:1-AR24:1 along with AR16:1*n-9* and AR26:1*n-9* being the sole isomer, respectively (Table 2).

**Fig. 7:**
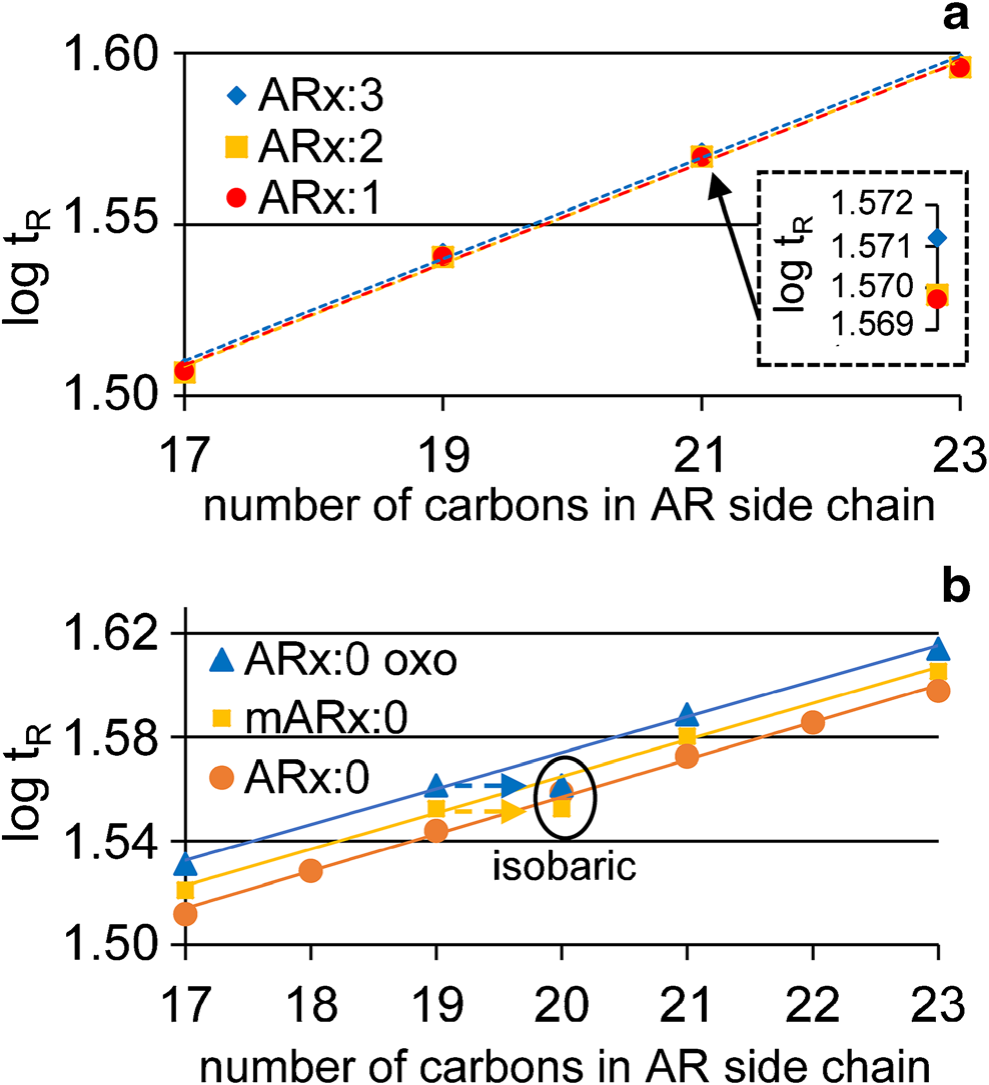
Plots of the logarithmic retention time against the carbon number in the alkyl chain of (**a**) triunsaturated ARs (ARx:3, *y* = 0.0148x + 1.2589, *R*^2^ = 0.9968), diunsaturated ARs (ARx:2, *y* = 0.0148x + 1.257, *R*^2^ = 0.9968) and monounsaturated *n-9* ARs (ARx:1, *y* = 0.0147x + 1.259, *R*^2^ = 0.9970), and (**b**) saturated ARs with a keto group (ARx:0 oxo, *y* = 0.0143x + 1.2882, *R*^2^ = 0.9988), saturated ARs (ARx:0, *y* = 0.0152x + 1.2544, *R*^2^ = 0.9987) and saturated mARs (mARx:0, *y* = 0.0148x + 1.2703, *R*^2^ = 0.9987). The correlation of the individual groups was calculated from 17 to 21 C atoms in the alkyl chain based on the temperature program. Extrapolation of AR19:0 oxo and mAR19:0 was plotted to isobaric saturated AR20:0

Diunsaturated ARs were generally odd-numbered and featured only one isomer, respectively, from AR17:2 to AR27:2 (Table 2). AR17:2, AR19:2 and AR21:2 contributed with 0.2–0.5%, respectively, to total ARs, whereas the others were only found in traces (Table 2). In all six occasions, the diunsaturated homologue almost co-eluted (GC) with the first eluting monounsaturated isomer (Δ*t*_R_ = 0.01–0.03 min, Table 2). This indicated that also double bonds of diunsaturated odd-chain ARs were located in the same positions (Table 2). Plots of log *t*_R_ against the carbon number in the alkyl chain resulted in stacked straight lines for diunsaturated ARn:2 and monounsaturated ARn:1*n-9* homologues (Fig. 7a). Suzuki et al. described the presence of *cis*-configured *n-6*- and *n-9*-double bonds in ARs [41].

Similar to the other ARs, diunsaturated ARs formed two DMDS adducts which differed by 46 u in mass. Interpretation of the mass spectra obtained from DMDS adducts in molecules with two nearby double bonds (here, methylene-interrupted) was found to be equivocal due to the possible formation of heterocyclic thietane, tetrahydrothiophene and tetrahydrothiopyran structures (4-, 5- and 6-membered rings) [42]. In the present case, however, the position of the terminal double bond at *n-6*-position could be verified by means of *m*/*z* 131 (*m*/*z* 61 + 5 • 14) (ESM Fig. S5). In addition, the position of the second double bond of AR21:2 ([C_36_H_68_S_4_Si_2_O_2_]^+^) could be assigned to *n-9*-position by means of *m*/*z* 513 ([C_26_H_49_S_2_Si_2_O_2_]^+^) in DMDS adduct 1 (ESM Fig. S5). In contrast to *m*/*z* 131, this fragment ion increases by *m*/*z* 513±14 • *n* with increasing chain length of the AR. The close relationship of GC retention times with the first eluting monounsaturated isomer (see above) indicated that all diunsaturated ARs featured the double bonds in *n-6*- and *n-9*-positions, respectively (Fig. 7a).

Finally, CCC fractionation enabled the detection of traces of five odd-numbered triunsaturated ARs including AR17:3 to AR23:3 (previously described in rye [6]) along with AR25:3 (Table 2). Unfortunately, positions and configurations of the double bonds could not be determined with the DMDS adduct method (data not shown). The triunsaturated ARs eluted 0.12 min after the corresponding diunsaturated ARs from the GC/MS column (exception AR25:3, due to final oven temperature). Plots of log *t*_R_ against the carbon number in the alkyl chain resulted in straight lines for triunsaturated ARs, which was different from the abovementioned di- and monounsaturated ARs (Fig. 7a). This also produced strong evidence that the double bonds of all homologues were always in the same positions (counted from the tail end).

In CCC, the elutions of the unsaturated ARs followed the abovementioned ECL rule (one double bond reduced the CCC elution volume by two carbons). Accordingly, AR17:0, AR19:1, AR21:2 and AR23:3 were found in the same elution range (Fig. 4). However, in dependence of the amount, ARs were present in more or less CCC fractions. ARs found in traces were only detected in one CCC fraction while abundant ARs were eluting in ten or more CCC fractions (Fig. 4). In addition, the more double bonds were present in a compound, the weaker the effect of the ECL, so a slight separation of the ECL pairs was possible (recognizable by the respective main fractions of the compounds) (Fig. 4, Table 2).

### Alkylresorcinols with keto groups in the alkyl chain

ARs with a keto group (oxo-ARs) showed higher GC *t*_R_ than the isobaric (saturated) ARs. Hence, both groups of ARs could be distinguished from each other [7]. Also, plots of log *t*_R_ against the carbon number in the alkyl chain resulted in straight lines for both saturated ARs and oxo-ARs (Fig. 7b). Extrapolation of the oxo-ARs to isobaric saturated ones (exemplarily shown in Fig. 7b for AR20:0 and AR19:0 oxo) verified constantly higher GC retention times of oxo-ARs. Seitz et al. assigned the keto group of oxo-ARs to β-position (i.e. C2 on the alkyl chain) which corresponds with a series of 5-(2-oxoalk(en)yl)resorcinols [7]. Apparently, oxo-ARs were formed by β-oxidation. The pattern of odd-numbered oxo-ARs from *R* = C_17_-C_25_ (4/50/28/15/5; values rounded) was similar but not in full agreement with the corresponding saturated ARs (27/33/29/8/3; values rounded).

Despite the observed differences in GC retention times, an even better distinction was possible by the distinctly different CCC elution profiles of oxo-ARs and isobaric *n*-alkyl-ARs. The higher polarity of oxo-ARs strongly accelerated their CCC elution compared with conventional ARs with the same alkyl length. Consequently, oxo-ARs eluted many CCC fractions earlier than the isobaric conventional ARs (which corresponded with a compensatory effect of ~ 5 additional carbon atoms or three double bonds. For example, the main CCC fraction of AR23:0 oxo (CCC fraction 14) was also the main CCC fraction of AR18:0 and AR23:3 (Fig. 4). Hence, CCC fractionation could provide equivocal information on the structures of AR families. This became also apparent by the detection of traces of oxo-ARs with one and two double bonds (Table 2). GC/MS spectra of trimethylsilylated oxo-ARs did not differ essentially from those of isobaric ARs without an oxygen, irrespective of the degree of saturation (e.g. AR21:0 oxo~AR22:0, AR21:1 oxo~AR22:1) (Fig. 5). The differentiation between unsaturated oxo-ARs and isobaric conventional ARs was possible due to the accelerated CCC elution of the oxo-ARs. Again, it was likely that unsaturated oxo-ARs were formed by β-oxidation. Verification of the oxo-group in β-position can be determined by analysis of free [7] or acetylated oxo-ARs. Free β-oxo-ARs show the McLafferty ion at *m*/*z* 166 which is formed by transfer of one H atom on C5 in the alkyl chain (in γ-position of the keto group) onto the oxygen of the carbonyl moiety followed by cleavage between C3 and C4 in the alkyl chain (ESM Fig. S6a). This fragment ion was also observed in di-acetylated oxo-AR at *m*/*z* 166 (additional removal of both acetyl groups) along with *m*/*z* 208 (additional removal of one acetyl group) (ESM Fig. S6b). In addition, α-cleavage on the left hand side of the keto group with the charge remaining in the alkyl chain resulted in the diagnostic fragment ions which additionally indicated the alkyl length of the oxo-AR (e.g. free and acetylated AR21:0 oxo produced *m*/*z* 295 (ESM Fig. S6). Di-fold acetylation shifted M^+^ of the free AR21:0 oxo (*m*/*z* 418) to *m*/*z* 502 (ESM Fig. S6). However, GC/MS spectra of acetylated oxo-ARs also featured ([M-84]^+^) and *m*/*z* 460 ([M-42]^+^) which corresponded with neutral loss of two and one acetyl group(s), respectively (ESM Fig. S6).

Again, the connection between conventional and β-oxidized unsaturated ARs was obvious: three monounsaturated oxo-ARs and one diunsaturated oxo-AR isomer were detected. In agreement with that, Suzuki reported *cis*-configured double bonds in *n-5*-, *n-7*- and *n-9*-positions for ARn:1 oxo and in *n-6*- and *n-9*-position in ARn:2 oxo [10] (Table 2). DMDS adducts of ARn:1 oxo confirmed these positions by means of the *m*/*z* series including the tail of the side chain at *m*/*z* 61+ *m* • 14 ([H(CH_2_)_*m*_CH=SCH_3_)]^+^) and *m*/*z* 387 + *n* • 14 ([CH_3_S=CH(CH_2_)_*n*_COC_6_H_2_SCH_3_(OSi(CH_3_)_3_)_2_]^+^) in DMDS adduct 1 for fragment ions including the head group (TMS resorcinol ring).

CCC fractionation and subsequent GC/MS analysis verified the presence of the known oxo-ARs in rye (i.e. AR19:0 oxo, AR21:0 oxo, AR23:0 oxo, AR25:0 oxo plus three AR21:1 oxo and three AR23:1 oxo isomers) along with three AR19:1 oxo isomers and AR17:0 oxo which were described for the first time in rye (Table 2). All four oxo-ARs were detected in CCC fractions 5 and 6, i.e. the first fractions containing ARs (Fig. 4). Likewise, the proposed ECL of conventional ARs was also valid for oxo-ARs (Table 2). In support of this finding, the diunsaturated AR21:2 oxo was also detected in CCC fraction 6 (Fig. 4), while AR23:2 oxo, AR21:1 oxo and AR19:0 oxo were detected in CCC fraction 8 (Table 2).

### Methylated alkylresorcinols with saturated alkyl chains

Several CCC fractions featured two saturated AR isomers (the same molecular ion). For instance, the classic AR22:0 (EV 96.6–129.2%) was accompanied with an “AR22:0” isomer showing an EV of 99.6–126.3%. The GC retention time of the second “AR22:0” isomer was shorter than the one of AR22:0 but GC/MS spectra were virtually identical except that the base peak of the “AR22:0” isomer was shifted by 14 u to higher mass (*m*/*z* 282 instead of *m*/*z* 268) (Fig. 5a, e). Since the base peak is formed via phenyl cleavage, stabilized by loss of one H atom, the additional “–CH_2_–” moiety must be either on the phenyl ring or in α-position of the alkyl chain. Such ARs were not reported before in rye. However, ARs with a further methyl group on the resorcinol ring were detected before in quinoa (mAR17:0-mAR26:0 and mAR23:1) and wheat leaf cuticle wax (odd-numbered mAR19:0-mAR27:0) [30, 43]. NMR analysis allowed to assign the methyl group to 2-position on the resorcinol backbone (i.e. between the two hydroxyl groups) [30]. Hence, it was most likely that the new ARs in rye also bore a 2-methyl substituent. Characteristic GC retention times and the diagnostic base peak at *m*/*z* 282 (see above and [30]) allowed to detect traces of five odd-chained mARs in the rye sample, namely mAR15:0-mAR23:0 (Table 2). mARs eluted together with the corresponding saturated AR isomer into the same CCC fractions, e.g. AR18:0 with mAR17:0 (Fig. 4, Table 2). Plots of log *t*_R_ against the carbon number in the alkyl side chain resulted in straight lines for mARs as well as the abovementioned saturated ARs and oxo-ARs (Fig. 7b). Again, these plots proved to be conducive to distinguishing members of different substance classes with similar GC/MS data (Fig. 7b). Extrapolation of the mARs to isobaric saturated ones (exemplarily shown in Fig. 7b for AR20:0 and mAR19:0) showed the shorter GC retention times of mARs. Accordingly, GC mARs eluted between the corresponding ARs with the same chain length on the one side and isomeric ARs, for instance AR17:0 < mAR17:0 < AR18:0.

### Concluding remarks

The presented method of combining CCC fractionation with offline GC/MS-SIM measurements strongly increased the number of detectable ARs. While GC/MS in full scan mode allowed the detection of eleven ARs, GC/MS-SIM enabled the detection of 29 ARs and CCC in combination with GC/MS-SIM allowed to detect 74 ARs in the rye sample. On the one hand, this approach helped to detect especially low abundant ARs. On the other hand, the orthogonal separation characteristics and especially the preparative nature of CCC made it possible to study uncommon ARs in CCC fractions by alternative methods such as DMDS adducts or acetylation. The use of a fraction collector and automated evaporator simplified the work load. However, the method is less suited for routine work as (here:) 80 fractions were analysed for one sample. In this context, other methods like LC/MS(MS) are more suited. Yet, the high diversity of ARs as determined in this study had not been reached before. In addition, the high sample load in CCC along with sample fractionation was crucial for the subsequent structure determination including derivatisation steps (e.g. by formation of DMDS adducts). This approach enabled the detection of several novel ARs in rye including odd-chained methylated ARs, even-numbered monounsaturated ARs, triunsaturated AR and saturated oxo-AR. The potential of this established method can now be used for profiling other matrices. In particular, the comparison of cereal species with each other, the search for further unknown ARs and the targeted isolation of individual ARs by CCC are potential areas of this methodology.

## Electronic supplementary material

ESM 1 (PDF 678 kb)
