# Supplementary material for: Countercurrent chromatographic fractionation followed by gas chromatography/mass spectrometry identification of alkylresorcinols in rye
Source: Anal Bioanal Chem. 2020 Oct 10;412(30):8417–30. doi: 10.1007/s00216-020-02980-3 (PMC7680747; doi:10.1007/s00216-020-02980-3)
Supplement: Supplementary file 1 — (PDF 678 kb) [file 216_2020_2980_MOESM1_ESM.pdf]

Analytical and Bioanalytical Chemistry

Electronic Supplementary Material

Countercurrent chromatographic fractionation followed by gas chromatography/mass spectrometry identification of alkylresorcinols in rye

Tim Hammerschick, Tim Wagner, Walter Vetter

Table S1 GC/MS-SIM measurement conditions with corresponding analytes

**Time window**	**Time period**	**Recorded ions**	**Analytes****
a	7-24 min	*m/z* 73, 313, 328, 335, 337, 339, 341, 350, 352, 454, 356	silylated free fatty acids FA16:0, FA18:0, FA18:1, FA18:2, FA18:3
b*	24-28 min	*m/z* 74, 87, 323, 339, 354	ISTD 22:0-ME
c*	28-29.1 min	*m/z* 442.3, 444.3, 446.3, 448.3, 450.3	silylated AR14:x, AR13:x oxo, mAR13:x
d*	29.1-30 min	*m/z* 456.3, 458.3 460.3, 462.3, 464.3	silylated AR15:x, AR14:x oxo, mAR14:x
e*	30-31.7 min	*m/z* 470.4, 472.4, 474.4, 476.4, 478.4	silylated AR16:x, AR15:x oxo, mAR15:x
f*	31.7-33 min	*m/z* 484.4, 486.4, 488.4, 490.4, 492.4	silylated AR17:x, AR16:x oxo, mAR16:x
g*	33-34.4 min	*m/z* 498.4, 500.4, 502.4, 504.4, 506.4	silylated AR18:x, AR17:x oxo, mAR17:x
h*	34.4-35.6 min	*m/z* 512.4, 514.4, 516.4, 518.4, 520.4	silylated AR19:x, AR18:x oxo, mAR18:x
i*	35.6-36.8 min	*m/z* 526.4, 528.4, 530.4, 532.4, 534.4	silylated AR20:x, AR19:x oxo, mAR19:x
j*	36.8-37.9 min	*m/z* 540.4, 542.4, 544.4, 546.4, 548.4	silylated AR21:x, AR20:x oxo, mAR20:x
k*	37.9-39.1 min	*m/z* 554.4, 556.4, 558.4, 560.4, 562.4	silylated AR22:x, AR21:x oxo, mAR21:x
l*	39.1-40.1 min	*m/z* 568.4, 570.4, 572.4, 574.4, 576.4	silylated AR23:x, AR22:x oxo, mAR22:x
m*	40.1-41.5 min	*m/z* 582.5, 584.5, 586.5, 588.5, 590.5	silylated AR24:x, AR23:x oxo, mAR23:x
n*	41.5-43 min	*m/z* 596.5, 598.5, 600.5, 602.5, 604.5	silylated AR25:x, AR24:x oxo, mAR24:x
o*	43-44.8 min	*m/z* 610.5, 612.5, 614.5, 616.5, 618.5	silylated AR26:x, AR25:x oxo, mAR25:x
p*	44.8-54.5 min	*m/z* 624.5, 626.5, 628.5, 630.5, 632.5	silylated AR27:x, AR26:x oxo, mAR26:x

**m/z* 267.1, 268.1, 281.1 and 282.1 were recorded from the second time window (b) onwards during the remaining run

** x = 0 – 4

Table S2 Important key fragment ions in GC/MS for determining the position of the double bonds of silylated monounsaturated ARs in DMDS adduct 1. Bold letters indicate which fragment ion pairs were detected

		n-3		*n-4*		*n-5*		*n-6*		*n-7*		*n-8*		*n-9*		*n-10*	
AR	M^+	A[a]	R[b]	A[a]	R[b]	A[a]	R[b]	A[a]	R[b]	A[a]	R[b]	A[a]	R[b]	A[a]	R[b]	A[a]	R[b]
AR15:1	602	89	513	103	499	**117**	**485**	131	471	**145**	**457**	159	443	173	429	187	415
AR16:1	616	89	527	103	513	117	499	131	485	**145**	**471**	159	457	**173**	**443**	187	429
AR17:1	630	89	541	103	527	**117**	**513**	131	499	**145**	**485**	159	471	**173**	**457**	187	443
AR18:1	644	89	555	103	541	117	527	131	513	**145**	**499**	159	485	**173**	**471**	187	457
AR19:1	658	89	569	103	555	**117**	**541**	131	527	**145**	**513**	159	499	**173**	**485**	187	471
AR20:1	672	89	583	103	569	117	555	131	541	**145**	**527**	159	513	**173**	**499**	187	485
AR21:1	686	89	597	103	583	**117**	**569**	131	555	**145**	**541**	159	527	**173**	**513**	187	499
AR22:1	700	89	611	103	597	117	583	131	569	**145**	**555**	159	541	**173**	**527**	187	513
AR23:1	714	89	625	103	611	**117**	**597**	131	583	**145**	**569**	159	555	**173**	**541**	187	527
AR24:1	728	89	639	103	625	117	611	131	597	**145**	**583**	159	569	**173**	**555**	187	541
AR25:1	742	89	653	103	639	**117**	**625**	131	611	**145**	**597**	159	583	**173**	**569**	187	555
AR26:1	756	89	667	103	653	117	639	131	625	145	611	159	597	**173**	**583**	187	569
AR27:1	770	89	681	103	667	**117**	**653**	131	639	**145**	**625**	159	611	**173**	**597**	187	583

[a] *m/z* values (61 + m • 14) for fragment ions including the terminal part of the alkyl chain ($[H(CH_2)_mCH{=}SCH_3]^+$)

[b] *m/z* values (359 + n • 14) for fragment ions including the silylated resorcinol moiety ($[CH_3S{=}CH(CH_2)_nC_6H_2SCH_3(OSi(CH_3)_3)_2]^+$)

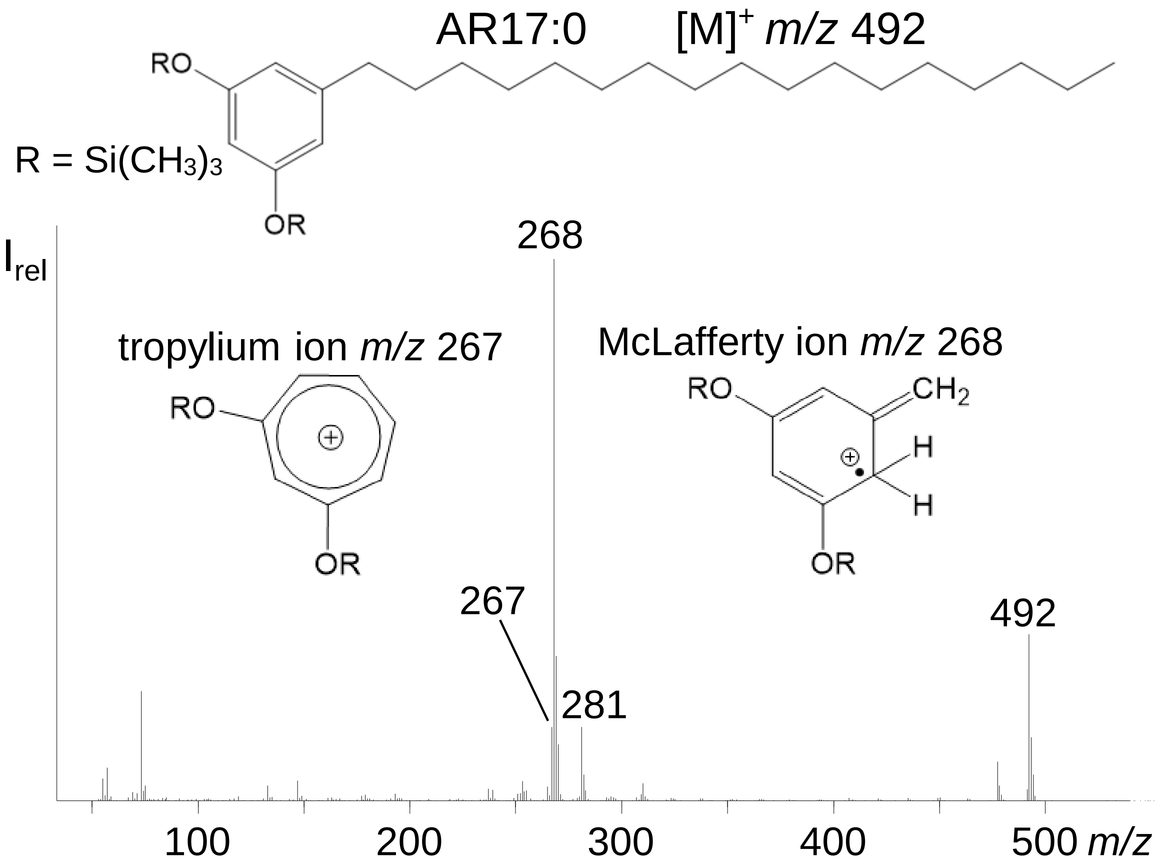

Fig. S1 Characteristic GC/MS fragment ions of silylated alkylresorcinols using the example of AR17:0

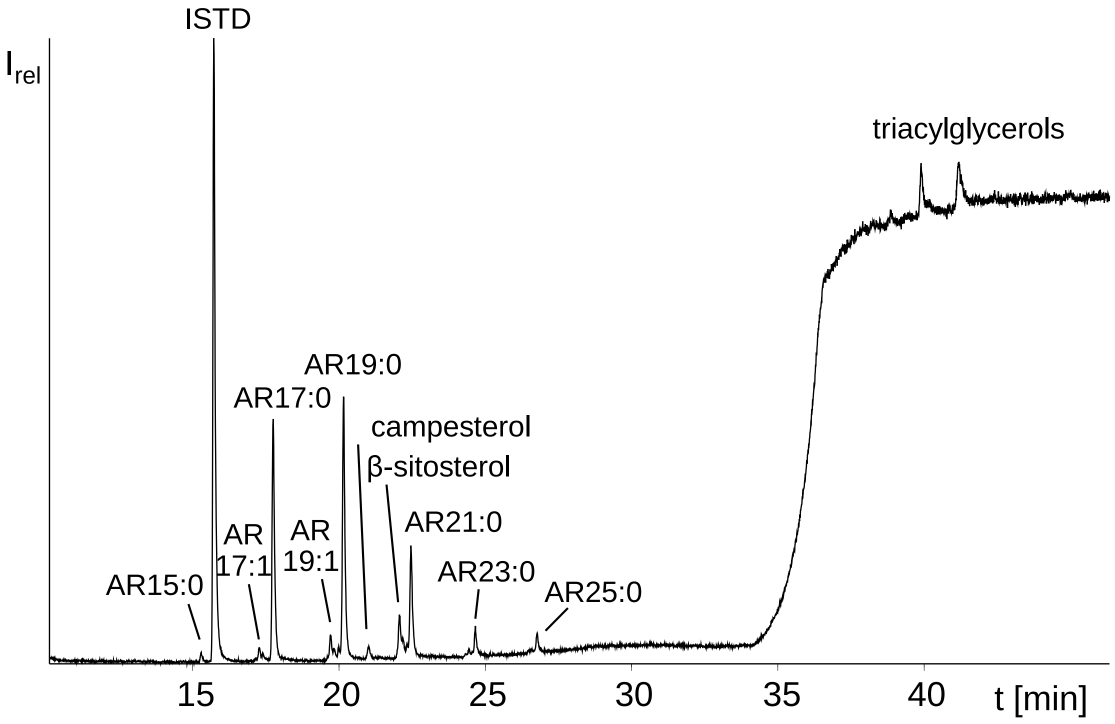

Fig. S2 GC/MS chromatogram (full scan mode) of silylated rye grain extract measured on GC/MS system 1 (ZB-1HT, 100 °C (1 min) – 10 °C/min – 250 °C (5 min) – 5 °C/min – 300 °C – 30 °C/min – 350 °C (10 min))

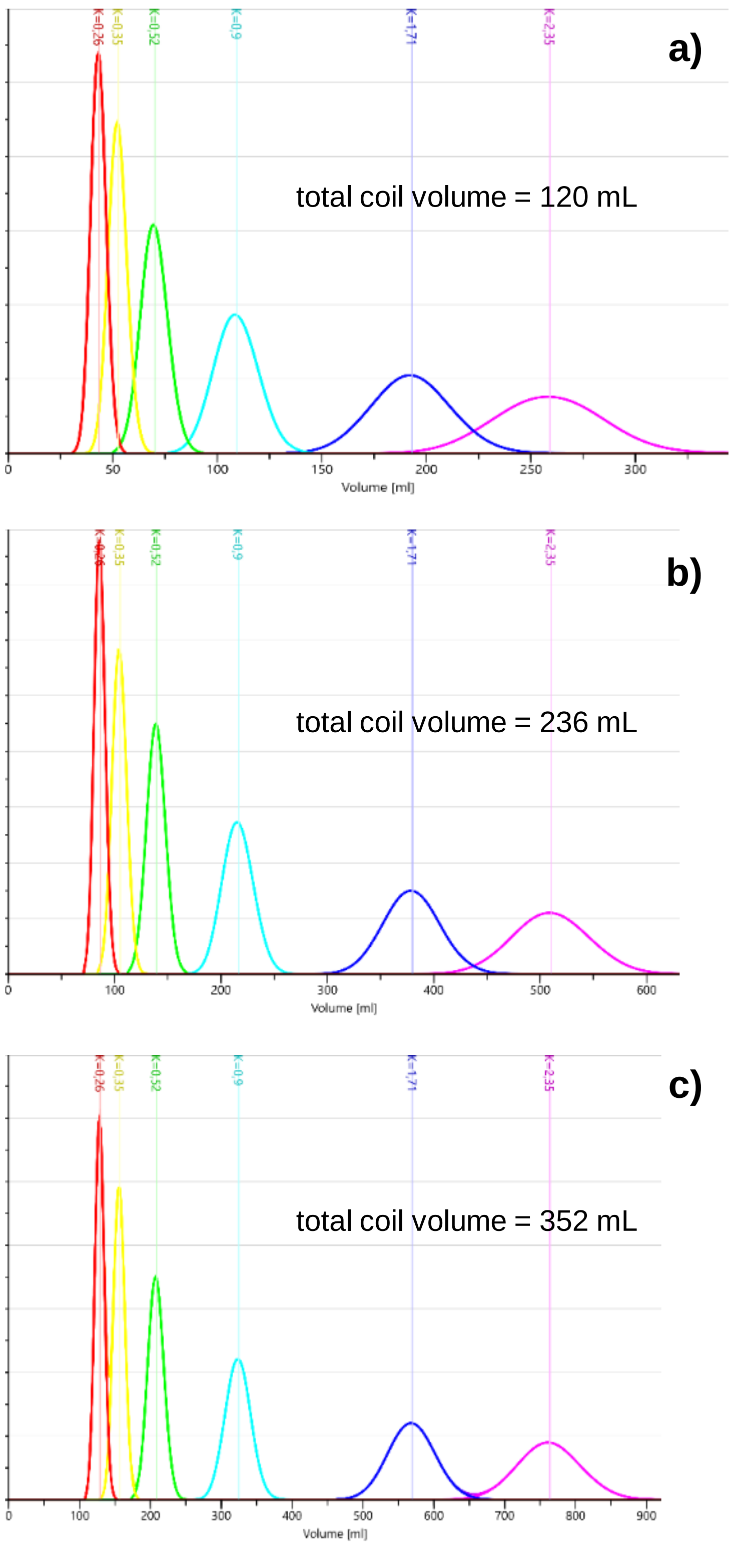

Fig. S3 Predicted elution volumes of AR15:0 (K=0.26), AR17:0 (K=0.35), AR19:0 (K=0.52), AR21:0 (K=0.9), AR23:0 (K=1.71) and AR25:0 (K=2.35) with a total coil volume of (**a**) 120 mL, (**b**) 236 mL and (**c**) 352 mL. The calculation with "ProMISE 2" was performed with S_f=86%, 2 mL/min flow, 870 RPM, efficiency 0.03, 10 mL injection volume in head-to-tail mode

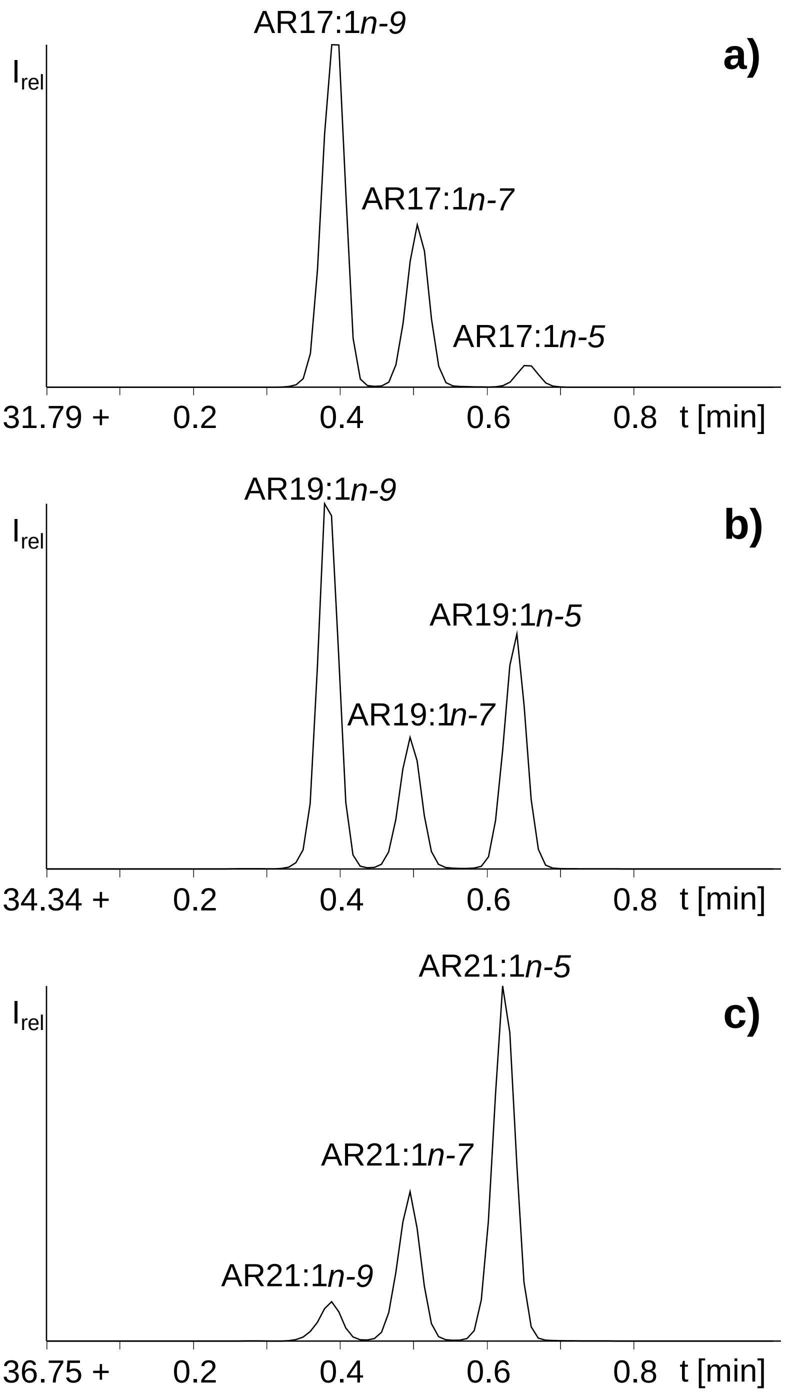

Fig. S4 GC/MS chromatograms (excerpts) containing the monounsaturated alkenylresorcinols of the silylated CCC fractions (**a**) 9, (**b**) 14 and (**c**) 22 (system 2, Optima 5HT, 55 °C (1 min) – 10 °C/min – 200 °C – 5 °C/min – 320 °C (15 min))

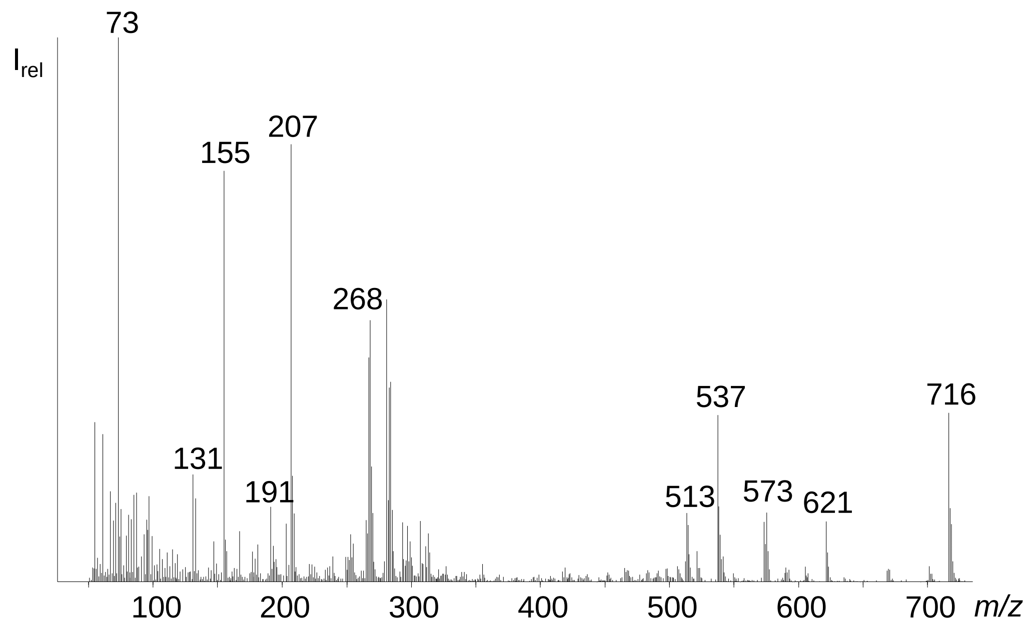

Fig. S5 GC/MS spectrum of the silylated DMDS adduct 1 of AR21:2 ($C_{36}H_{68}S_4Si_2O_2$)

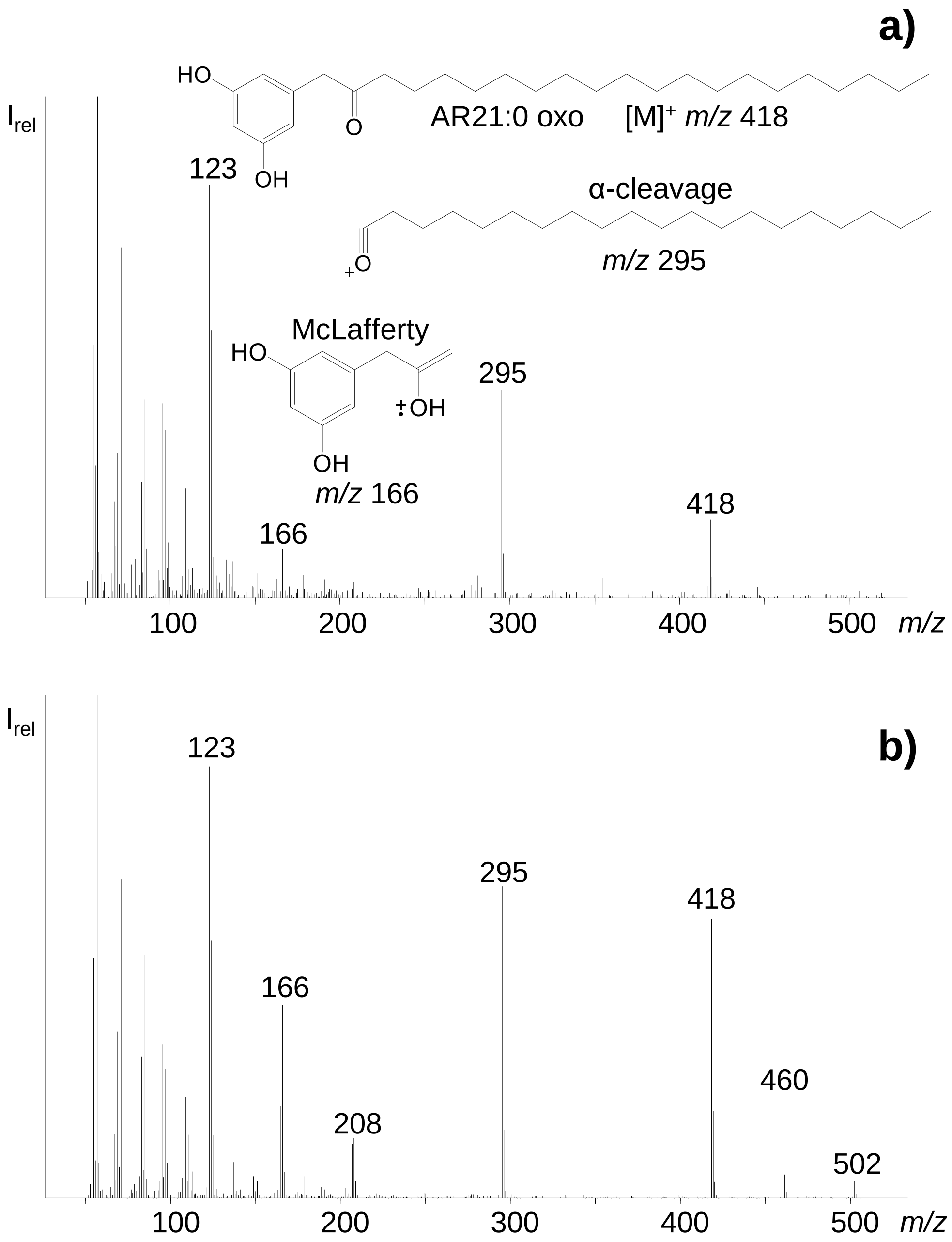

Fig. S6 GC/MS spectra of (**a**) AR21:0 oxo underivatized with the structures of the fragment ions produced by McLafferty re-arrangement and α-cleavage and (**b**) AR21:0 oxo acetylated
